# Supplementary material for: Absorptive pinhole collimators for ballistic Dirac fermions in graphene
Source: Nat Commun. 2017 May 15;8:15418. doi: 10.1038/ncomms15418 (PMC5440660; doi:10.1038/ncomms15418)
Supplement: Supplementary Information — Supplementary Figures, Supplementary Notes and Supplementary References [file ncomms15418-s1.pdf]

Supplementary Note 1

Classical ballistic conductance calculation

For most electron densities covered in the main text, electrons can be viewed as classical point-like particles whose momentum distribution is determined by the band-structure and Fermi-Dirac statistics. Similar to the calculation of current through 1D channels or point contacts [1], our type of collimator can be treated as a finite constriction connecting two electron reservoirs. Net current only flows in an energy band for which there is a Fermi energy mismatch $\Delta E = E_{f_2} - E_{f_1}$, where E_{f_1} and E_{f_2} are the Fermi energies of each reservoir. In two dimensions, the angular flux density is:

$$\frac{dJ}{d\theta} = \frac{e}{\hbar}\frac{d^2n}{d\theta dk}\Delta E \tag{1}$$

where n is the electron density, e is the charge of an electron, k is the electron wave vector magnitude, and θ is the angle of electron propagation. At the Fermi level,

$$\frac{d^2n}{d\theta dk} = \frac{g_k k_f}{4\pi^2} \tag{2}$$

where g_k is the degeneracy of a fixed k state. In the case of graphene, the degeneracy is 4 due to the valley and spin degrees of freedom. This gives:

$$\frac{dJ}{d\theta} = \frac{2e^2 k_f}{\pi h} V_{\text{bias}} = \frac{2e^2}{h}\sqrt{\frac{n}{\pi}} V_{\text{bias}} \tag{3}$$

where $V_{\text{bias}} = \Delta E/e$ is the voltage difference between reservoirs. The electron flux density is uniform and controlled by the 2D electron density. To calculate the angular current density $I(\theta)$, the width of the constriction at a given angle $w(\theta)$ must be known. For the case of the double pin-hole collimator, the projected width is $w(\theta) = \cos(\theta)\left[w_0 - L_0 \left|\tan(\theta)\right|\right]$ in the range $|\theta| < \tan^{-1} w_0/L_0$ where w_0 is the width of an aperture and L_0 is the separation between apertures. This means that the current emitted over a narrow range of angles $\Delta\theta$ is:

$$I(\theta)\Delta\theta = V_{\text{bias}}\frac{2e^2}{h}\sqrt{\frac{n}{\pi}}\cos(\theta)\left[w_0 - L_0\left|\tan(\theta)\right|\right]\Delta\theta. \tag{4}$$

In our angular distribution measurement, we collect ballistic electrons across the width of the device W_{dev} through a third pinhole collector as we vary the magnetic field. The collector aperture has an acceptance angle in the small angle approximation that goes as $\Delta\theta \approx \frac{w_0 \cos(\theta)}{W_{\text{dev}}}$. Convolving the injection angular distribution with the collector angular distribution results in the predicted nonlocal conductance:

$$G = \frac{2e^2}{h}\sqrt{\frac{n}{\pi}}\int_{\theta_B - \frac{w_0}{2W_{\text{dev}}}\cos(\theta_B)}^{\theta_B + \frac{w_0}{2W_{\text{dev}}}\cos(\theta_B)} d\theta \cos(\theta)\left[w_0 - L_0\left|\tan(\theta)\right|\right] \tag{5}$$

where $\theta_B = \sin^{-1}\left(\frac{eB}{h}\sqrt{\frac{\pi}{n}}W_{\text{dev}}\right)$ is the central angle injected that reaches across the width of the device at a given B. We integrate numerically Supplementary Eqn. 5 with $w_0 = 300$ nm and $L_0 = \{0 \text{ nm}, 850 \text{ nm}\}$ to produce the plot in Fig. 1d in the main text. If the width of the collector is sufficiently small, Supplementary Eqn. 5 reduces to:

$$G(\theta_B) = \frac{2e^2}{h}\sqrt{\frac{n}{\pi}}\frac{w_0}{W_{\text{dev}}}\cos^2(\theta_B)\left[w_0 - L_0\left|\tan(\theta_B)\right|\right] \tag{6}$$

The full width at half maximum then comes from solving: $\cos^2\frac{\theta_{\text{FWHM}}}{2}\left[1 - \frac{L_0}{w_0}\tan\frac{\theta_{\text{FWHM}}}{2}\right] = \frac{1}{2}$. For narrow beams, this simplifies to: $\theta_{\text{FWHM}} = \frac{w_0}{L_0}$.

Supplementary Note 2

Transverse electron focusing

Unlike in traditional transverse electron focusing (TEF) measurements, the center of most cyclotron orbits exiting our double-pinhole collimators are *not* collinear with the device edge. Instead, the median center is displaced off the edge by $L_0/2$, and as a result, successive focusing peaks occur at irrational ratios. For an injector-collector separation X_0, the radii of curvature that result in peak conduction from the collimator to the adjacent collector follow the relationship:

$$r_n = \sqrt{\left(\frac{L_0}{2}\right)^2 + \left(\frac{X_0}{2n}\right)^2} \tag{7}$$

In our collimating device, $X_0 = 2.3\,\mu\text{m}$ and $L_0 = 850\,\text{nm}$. Two related consequences of the arc center offset are (1) the incident angle to edges is no longer normal ($\theta = \frac{\pi}{2}$) but rather $\theta_n = \tan^{-1}\frac{L_0}{nX_0}$ and (2) there is a fixed minimum radius $r_\infty = \frac{L_0}{2}$ below which no ballistic conduction should occur.

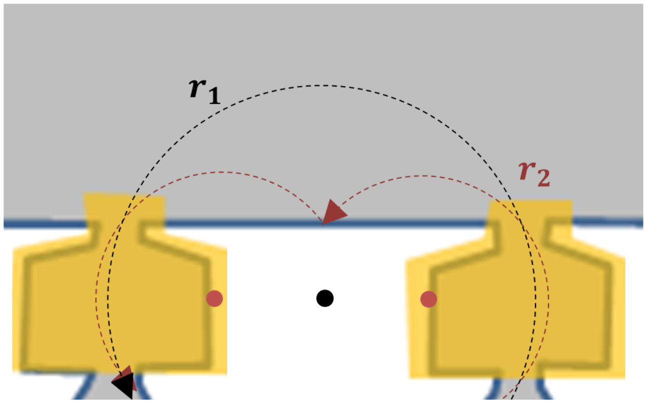

Supplementary Figure 1: diagram of TEF trajectories in our collimators. The center of each arc is plotted as a solid circle, and is offset from the edge by half the length of a collimator. The consequence of this: TEF peaks are not evenly spaced and the incident angle varies with r_n.

Supplementary Note 3

Diffraction effects on transport: 2D Dirac equation simulations

Low energy electron excitations in graphene obey the 2D massless Dirac equation. This behavior implies that electrons in our collimators should be subject to diffraction, which may particularly affect electron transport at low electron densities. We thus perform finite-difference time-domain (FDTD) simulations of the Dirac equation to predict the transport behavior of our collimators.

The Dirac equation for electrons in graphene is:

$$\pm v_\text{f}\boldsymbol{\sigma}\cdot\nabla\Psi = -\partial_t\Psi \tag{8}$$

where $\boldsymbol{\sigma}$ is a vector representation of the two Pauli matrices σ_x and σ_y, v_f is the Fermi velocity, ∂_t connotes a single partial time derivative, and ψ is the two-component single-particle wavefunction. Defining the two components as $\Psi \equiv \begin{pmatrix} u \\ v \end{pmatrix}$, and working in graphene's natural units ($\hbar = 1$ and $v_\text{f} = 1$), Supplementary Eqn. 8 can be expressed as two coupled equations:

$$\partial_t u \pm \partial_x v \mp i\partial_y v = 0 \tag{9}$$

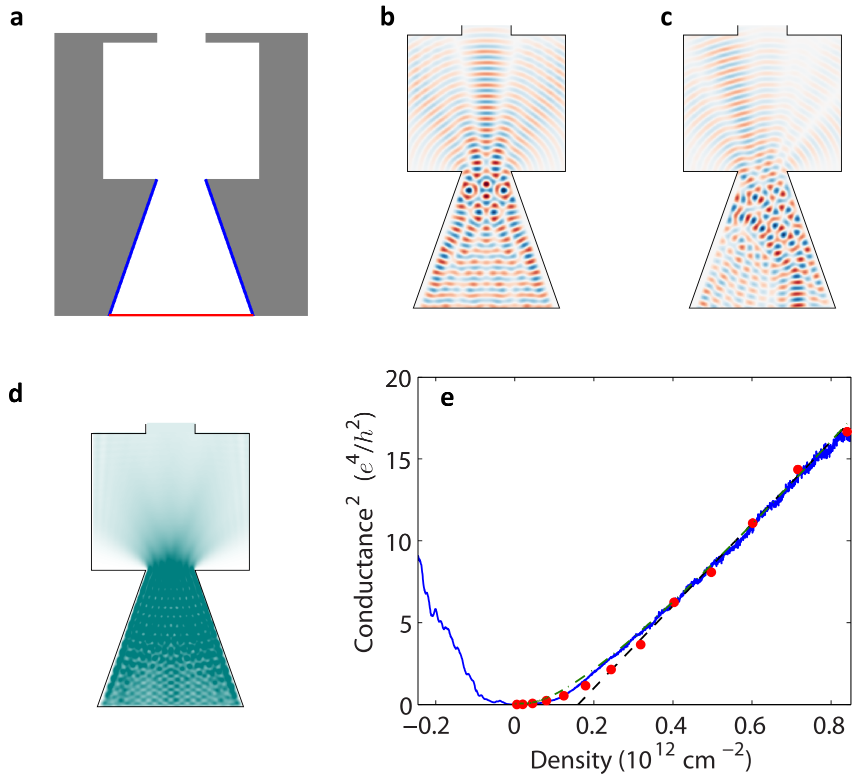

Supplementary Figure 2: **a)** Defined regions for simulation. gray corresponds to absorptive conditions, blue to reflective, and red to the injection region. **b)** real part of u for a single plane-wave solution. **c)** particular instance of random electron injection. **d)** Magnitude of J_y (the current density in the vertical direction). **e)** Same measurement data, simulation, and $\sqrt{n-n_0}$ fit as contained in Fig. 2a of the main text, with the y-axis squared to emphasize the square-root-dependence. Included is a phenomenological fit as a green dashed line.

$$\partial_t v \pm \partial_x u \pm i\partial_y u = 0 \tag{10}$$

We discretize Supplementary Eqns. 9 and 10 using a staggered space and staggered time approach[2] (which solves the fermion doubling problem), and apply reflective or absorptive boundary conditions. Reflective boundary conditions are employed by setting u to zero at boundaries and allowing v to propagate freely[3] (setting boundary conditions on u instead of v is arbitrary; the reverse selection would be equivalent). Absorptive boundaries are employed using the "absorbing potential" approach[2]. As a method of injecting electrons, we couple in a time-varying complex amplitude at the edge of the collimator ohmic contacts (Supplementary Fig. 2a). We inject plane-waves (Supplementary Fig. 2b) at several injection angles to be summed over later.

To calculate total conductance of the collimator, we iteratively sum the plane-waves solutions at a fixed energy with random amplitudes to approximate integration over all spatial probability amplitudes. During each iteration (Supplementary Fig. 2c) we compute the current density:

$$\mathbf{J} = ev_{\mathrm{f}}\Psi^{\dagger}\boldsymbol{\sigma}\Psi \tag{11}$$

and then average $\mathbf{J}$ over all iterations (Supplementary Fig. 2d). The conductance plotted in Fig. 2a (and Supplementary Fig. 2e) then results from integrating over $\mathbf{J}$ at the top aperture.

These simulations give an absolute prediction for the collimator conductance. However, as discussed in the main text, the measurement in Fig. 2a is partially attenuated by electron reflections and reabsorption by the filter ohmic. Thus, we allow one free parameter: the absolute

conductance. Good agreement between simulation and experiment is evident from the proper scaling of the conductance with density: namely, in the limit of large n, the conductance goes as $G \sim \sqrt{n - n_0}$. n_0 is not a free parameter of the fit, yet it agrees well with the data.

This effect is likely due to the reflective boundary conditions of the first pinhole, as well as diffraction throughout the collimator. At density n_0, the Fermi wavelength $\lambda_\mathrm{f} = 89$ nm is an appreciable fraction of the width of the slits; under these conditions, the beam diffracts significantly off of itself, constraining the available spatial modes that fit between slits. While a detailed analysis of the mode shapes is beyond the scope of this work, it is worth noting that the Fraunhofer diffraction off a single slit with this low density only has $\theta_\mathrm{FWHM} \approx 17°$, which is still narrower than the classical angular distribution discussed above. This means that even under modest doping, diffraction does not appreciably broaden the collimated beam.

A way to phenomenologically parametrize the net effect of diffraction and interference is to assert that the effective width of the apertures is reduced by a length proportional to λ_f. We find that making the mapping $w_0 \to w_0 - \lambda_\mathrm{f}$ in Eqn. 2 of the main text reproduces our data well. With this ansatz, we fit our data with one free scaling parameter (Supplementary Fig. 2e) and find excellent agreement. The generality of this exact mapping for different collimator geometries bears further investigation, however the basic observation that finite-wavelength effects moderately reduce the conductance relative to classical ballistic theory seems robust.

Supplementary Note 4

Comparison of simulation to existing methods

Our approach is built on the intuition that the ohmic leads should be a source of electrons that are in superpositions of plane-wave states: since we are not factoring in any information on the shape of the lead, we chose to treat the lead as a half-plane[4]. Our approach is valid in the single-particle limit and the linear response regime, however other approaches, such as the non-equilibrium Green's function (NEGF) technique could be applied to the system to include considerations of scattering and application of higher biases.

In a typical NEGF approach, semi-infinite leads are coupled to a finite region of interest, an orthogonal basis of input and output conduction channels are solved for, and the system's Green's function is computed in a manner that connects the finite region to these channels via a "lead self energy". With the solved Green's function, the total transmission or current density can be determined. In the Landauer-Büttiker formalism, which generally describes phase-coherent transport including in the NEGF approach, each conduction channel caries a maximum conductance $\frac{e^2}{h}$. With this in mind, a worthwhile test of our approach is to show that our injection scheme evenly populates the transverse modes of a fictitious semi-infinite lead.

To that end, we compute several instances of superposed, equiangularly-spaced plane-wave states, and Fourier decompose the wave function $\psi(x, z)$ at $x = 0$ and $0 < z < W$ as a means of determining the drive amplitude of transverse modes of a semi-infinite lead $\chi_n \propto \sin \frac{n\pi z}{W}$. The apparent amplitude increases at high n (Supplementary Fig. 3a). However, the group velocity $v_{\mathrm{g}n} = v_\mathrm{f} \frac{k_{zn}}{k_\mathrm{f}}$ of transverse waveguide modes (Supplementary Fig. 3b) is proportionally reduced at high n so that the total transmission of each mode is constant (Supplementary Fig. 3c). The abrupt cut-off for $n > \frac{k_\mathrm{f} W}{2\pi}$ comes from the absence of any Fourier components greater than k_f.

This approach trades elegance and efficiency for conceptual simplicity. Our injection scheme doesn't require basis transformation or matrix inversion: we simply define a geometry, inject at several different plane-wave angles and then create random superpositions of these states to stochastically solve for current and charge densities.

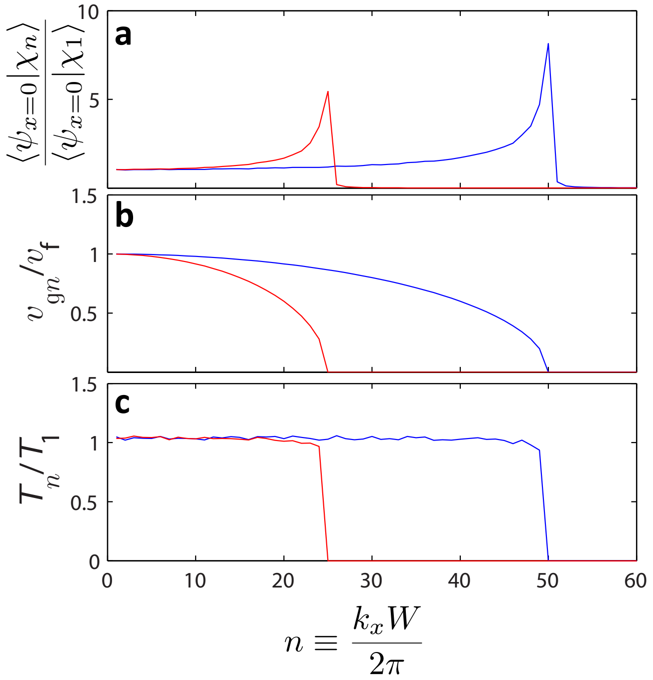

Supplementary Figure 3: Transverse mode occupation of simulated injection scheme at two Fermi wavelengths. **a)** normalized Fourier decomposition of the 1D wave amplitude at of a finite width W aperture with $k_\mathrm{f}W = 2\pi\times25$ (red) and $k_\mathrm{f}W = 2\pi\times50$ (blue). **b)** longitudinal group velocity of transverse modes. **c)** normalized transmission in each transverse mode taken as a product of **(a)** and **(b)** to reflect total conductance. ***The uniform population up to*** $n >= \frac{k_\mathrm{f}W}{2\pi}$ ***indicates that our scheme properly excites all transverse modes*** up to a normalization factor. In **a-c**, 10^4 random instances of the mixed plane-wave state were averaged.

Supplementary Note 5

Background subtraction for determining transmission probability

To accurately measure the transmission probability of electron trajectories through our collimators, we measured a doubly-collimated signal. We found that this significantly reduces the impact of a diffuse background, however, a small background remains even in this configuration. Here, we discuss our approach regarding background subtraction. There is insufficient information to fully disentangle the contributions of ballistic transport and the diffuse background, so we instead compute upper and lower bounds for the background.

For the upper-bound, we first observe that the wide-collection angle signal (S3F3) has a nearly identical functional form for the diffuse background as for the narrow collection angle signal (S3). We plot S3F3/9 over S3 in Supplementary Fig. 4, and observe significant agreement between the two curves at $|B| > 50$ mT. If we assume that the majority of the background current in S3 results from electrons scattering inside the collimator chamber, then the background is simply S3F3/9. We plot S3-S3F3/9 (solid black line, Supplementary Fig. 4) which has a peak height of 0.042.

Alternatively, if we assume that the contacts in F3 absorb all incoming electrons, then the only available background electrons must pass ballistically through both collimator apertures. For simplicity, if we assume an isotropic background, the background should be proportional to $\cos^2\theta$, where $\theta = \sin^{-1}\left(\frac{eB}{h}\sqrt{\frac{\pi}{n}}L_0\right)$. We fit such a curve to the regime $|B| > 50$ mT (dotted black line, Supplementary Fig. 4). Using this background, the conductance peak height is 0.051.

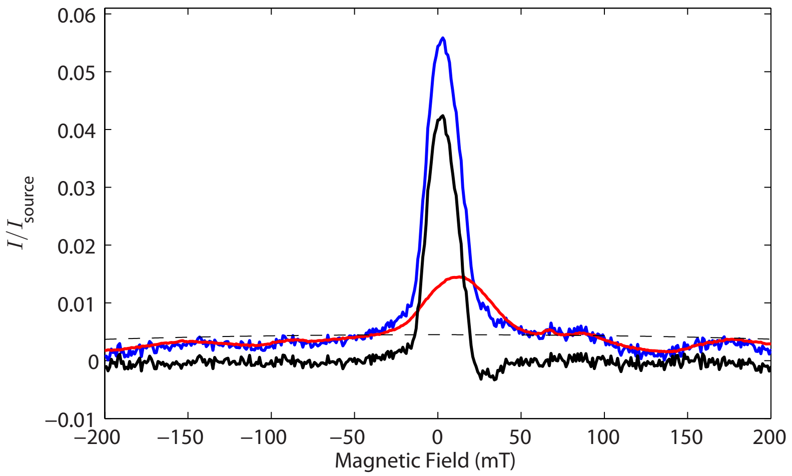

Supplementary Figure 4: Diffuse background subtraction for measuring transmission probability. Normalized current S3 is plotted in blue. The normalize current S3F3 is scaled by a factor of 9 to best fit the diffuse background of S3 (red line). The difference of the two, S3-(S3F3)/9, is plotted as solid black. This represents a lower-bound for the background-subtracted conductance. Alternatively, a fit assuming a completely isotropic background is plotted as a dotted black line. This should be the minimum background signal.

The true diffusive background is likely between these two limits given that the contacts have reasonably high (but not perfect) transmission probabilities.

Supplementary Note 6

Estimate of attainable collimator currents

In the main text's discussion pertaining to Fig. 2, we sourced $I_{\text{source}} = 50\text{ nA} \lesssim \frac{k_{\text{B}}T}{\text{e}R_{\text{source}}}$, where $R_{\text{source}} \sim 1\text{ k}\Omega$ is the resistance from the source to all other contacts. The choice to operate in this low injection current regime ensures that the applied bias is below the thermal bandwidth ($k_{\text{B}}T = 140\ \mu\text{eV}$) at our measurement temperature of $T = 1.6$ K, and that the measurement stays well within the linear response regime.

In our measurements, we did not probe the maximum current that could be sourced while maintaining collimation; however this is a relevant consideration for potential applications. With this in mind, ohmic contacts of the size we use can readily pass $20\times$ more current than in our measurement without degradation. If we were to increase the injected current to this level (1 μA), we would not expect substantial changes in the collimated beam shape: the thermal bandwidth would be significantly increased but would remain below 30 K. Recent work[5] has indicated that the mean free path at this energy scale would be $\sim 10\ \mu$m; this is large compared with the width of our device (2 μm), which means that ballistic transport should still dominate. Considering this, we anticipate that the relatively low current in the doubly-collimated configuration could be enhanced to ~ 60 nA without appreciable loss of collimation.

Supplementary Note 7

Ballistic simulations

Because most of the graphene sheet perimeter is not contacted by ohmics, many electrons scatter several times off of device boundaries before being absorbed by a grounded ohmic. This makes it

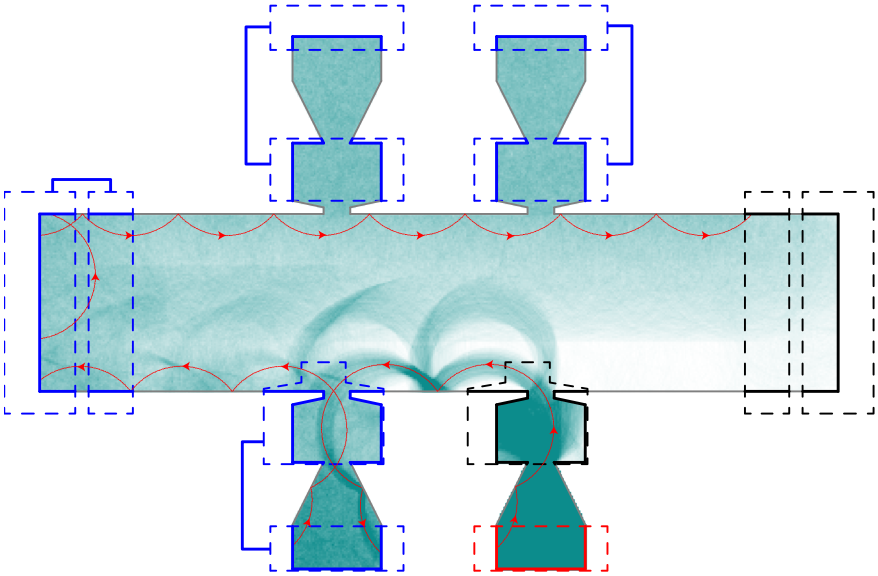

Supplementary Figure 5: A simulation of our Hall-bar at the first magnetic focusing peak ($r_c = 720\,\mathrm{nm}$) with $p_{\text{scatter}} = 0$ and $p_{\text{trans}} = 0.667$. Carriers are sourced at the red edge contact and flow through the device until they reach one of the grounded (black) contacts. Blue contacts are floating, and all dashed lines indicate physical contacts. One particular carrier's path is highlighted in red with arrows pointing in the direction of travel. Darker background color indicates higher density of trajectories.

important to consider all possible trajectories in accurately modeling our device. Charge carriers are first injected into the graphene sheet at a random position along the source contact and at a random angle. These carriers follow their classical trajectories until they hit an edge or they scatter in the bulk with a characteristic scattering length l_{scatter}. When they hit an edge, the appropriate behavior–scatter, specularly reflect, transmit–is determined from the nature of the edge hit and from control parameters p_{scatter}, the probability an edge will randomly scatter, and p_{trans}, the probability that an ohmic will transmit. Refractive behavior is not considered in this analysis given that the electron density is expected to be uniform over the bulk of the device. This process is repeated for each injected carrier until it is absorbed in a grounded contact. In the simulations both here and discussed in the main text, 40,000 carriers were injected into the sheet.

For current measurements, current drains are grounded and the number of carriers absorbed into each contact is recorded. The measured current flowing out a particular contact is given by $I_{\text{contact}}/I_{\text{source}} = n_{\text{contact}}/N$ where n_{contact} is the number of carriers that end up in a particular grounded contact and N is the total number of injected carriers. To ensure detailed-balance, floating voltage leads are simulated by absorbing incident carriers, followed by re-emitting the same carriers at random positions and angles. The voltage in these leads should be proportional to the flux of carriers through the edge contacts. Thus, the voltage is given by $V_{\text{meas}} \propto \phi_{\text{contact}}/L_{\text{contact}}$ where ϕ_{contact} is the number flux of carriers through the contact and L_{contact} is given by the length of the contact along the graphene sheet.

To model edge behavior in our device, we need to account for the complete fabricated device geometry. In particular, certain ohmics were mildly misaligned, resulting in asymmetric edge contact to the device region in C1 and C4. Based directly off of optical micrographs of the device (e.g. Fig. 1b), we defined the geometry and contacts as shown in Supplementary Fig. 5. The graphene sheet is defined first (grey), then contacts are added on top (dashed lines) in the measurement configuration from Fig. 3 of the main text. Shown is a snapshot at a constant

magnetic field corresponding to a radius of curvature of $r_c = 720\,\mathrm{nm}$. We have highlighted one trajectory as a guide to the eye. Note that this trajectory is absorbed and re-emitted several times by floating ohmics.

The scattering probability of the edges is an experimental unknown that we try to understand based on comparison with our simulations. We thus conducted magneto-transport simulations at several values of p_{scatter}, which is the probability that a given charge carrier will scatter following a cosine distribution normal to the edge, while holding $p_{\text{trans}} = 0.67$ constant. We compare the area under the first (no bounce) and second (one bounce) TEF peaks, and as seen in Supplementary Fig. 6, we simulated magnetic field sweeps over a range from $p_{\text{scatter}} = 0$ to $p_{\text{scatter}} = 1$. The second peak's area decreases linearly with increasing scattering probability while the first remains largely unchanged, so we compare the ratio with our experimental data both on the hole and electron side to find that the scattering probability on the electron side is $p_{\text{scatter}} \approx 1$, and $p_{\text{scatter}} \approx 0.5$ on the hole side.

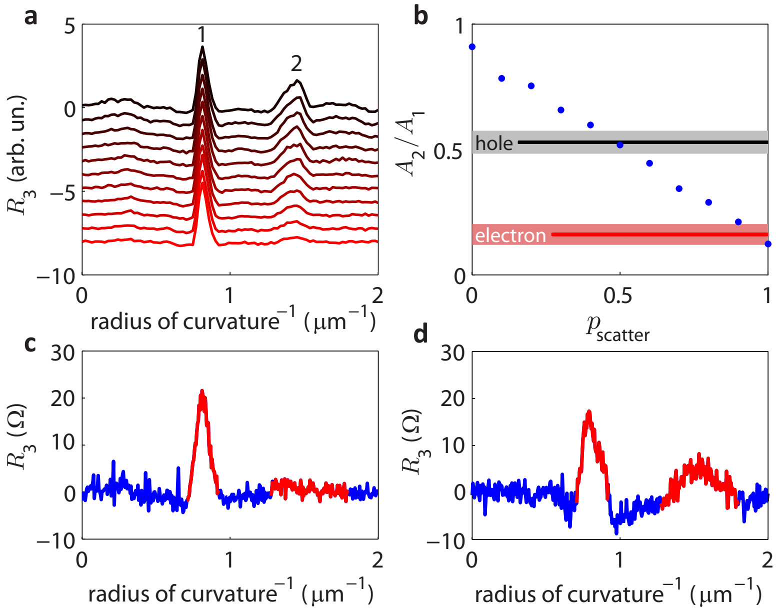

Supplementary Figure 6: Edge scattering simulations. **a)** Simulated magnetic field sweeps using the contact scheme shown in Supplementary Fig. 5. The edge scattering probabilities range from $p_{\text{scatter}} = 0$ (red) to $p_{\text{scatter}} = 1$ (black). There is little change in the first focusing peak (1), but the second peak (2) decreases in height and area with increasing scattering probability. **b)** Ratio of area under the second magnetic focusing peak to the area under the first decreases linearly with increasing edge scattering probability. The hole- and electron-side ratios for our data are plotted in black and red, respectively. The lighter-colored shading indicates the uncertainty in the peak ratios from our data. Measurement data for electron doped regime **(c)** and hole doped regime **(d)**. Integration ranges for peaks are highlighted in red.

To optimize p_{trans}, we observe the functional form of all three non-local resistances and find the best-fit conditions. For the electron side, this corresponds to $p_{\text{trans}} = 0.67$. For the hole side, the contact resistances are higher, and the best fit simulation is $p_{\text{scatter}} = 0.67$ and $p_{\text{trans}} = 0.1$. As is evident, the scattering probability is modestly higher than the above analysis, resulting from a weak dependence of A_2/A_1 on p_{trans}. We plot the final fit on the hole side (analogous to Fig. 3d in the main text) in Supplementary Fig. 7. The fit is qualitatively good, though not as striking as for the electron side. Given that there is substantially greater spurious emission due to high contact resistance as well as higher specularity of reflections for the hole-doped data, it is actually surprising to have as close a fit as observed.

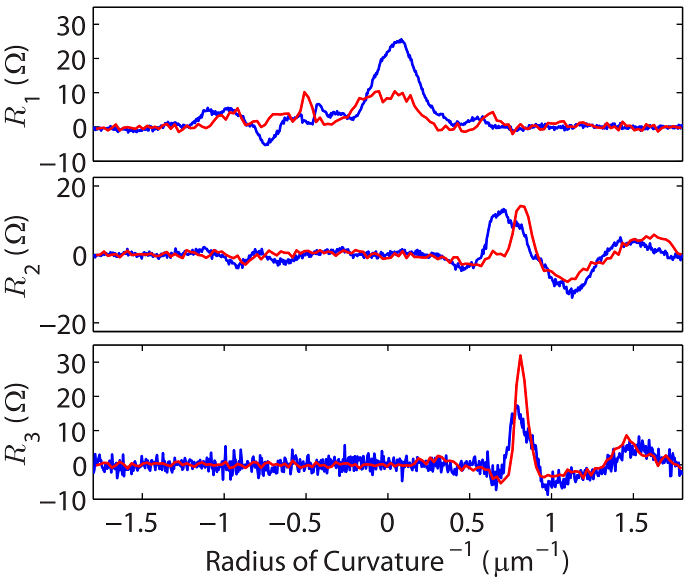

Supplementary Figure 7: Simulation vs experiment in hole doped regime. Data are plotted in blue, and simulation results ($p_{\text{scatter}} = 0.67$ and $p_{\text{trans}} = 0.1$) are plotted in red.

Supplementary References

[1] Ryndyk, D. Landauer-Büttiker Method. In *Theory of Quantum Transport at Nanoscale*, 17–54 (Springer International Publishing, 2016).

[2] Pinaud, O. Absorbing layers for the Dirac equation. *Journal of Computational Physics* **289**, 169–180 (2015).

[3] Alonso, V., Vincenzo, S. D. & Mondino, L. On the boundary conditions for the Dirac equation. *European Journal of Physics* **18**, 315 (1997).

[4] Barnett, A. H., Blaauboer, M., Mody, A. & Heller, E. J. Mesoscopic scattering in the half plane: Squeezing conductance through a small hole. *Physical Review B* **63**, 245312 (2001).

[5] Lee, M. *et al.* Ballistic miniband conduction in a graphene superlattice. *Science* **353**, 1526–1529 (2016).
